# Enhancing transition care for adolescents and young adults with adrenal insufficiency in the Netherlands: a holistic model for improved patient outcomes

**DOI:** 10.1530/EC-25-0324

**Published:** 2025-10-06

**Authors:** H L Claahsen-van der Grinten, K Davidse, K M A Dreijerink, J P van Eck, N Reisch, S Lajic, E Foltête, N Stikkelbroeck, H Vlaardingerbroek

**Affiliations:** ^1^ Radboud University Medical Centre, Amalia Childrens Hospital, Department of Pediatrics, Division of Pediatric Endocrinology, Nijmegen, The Netherlands; ^2^ Erasmus University Medical Centre, Department of Internal Medicine, Division of Endocrinology, Rotterdam, The Netherlands; ^3^ Amsterdam University Medical Centre, Department of Endocrinology and Metabolism, Amsterdam, The Netherlands; ^4^ Erasmus University Medical Centre, Department of Pediatrics, Division of Pediatric Endocrinology, Rotterdam, The Netherlands; ^5^ Medizinische Klinik IV, LMU Klinikum München, Munich, Germany; ^6^ Department of Women’s and Children’s Health, Karolinska Institutet, Department of Pediatric Endocrinology, Karolinska University Hospital, Stockholm, Sweden; ^7^ Department of Clinical Sciences, University of Gothenburg, Gothenburg, Sweden; ^8^ Bijniervereniging, NVACP, Haarlem, The Netherlands; ^9^ Radboud University Medical Centre, Department of Internal Medicine, Division of Endocrinology, Nijmegen, The Netherlands; ^10^ Willem-Alexander Children’s Hospital, Leiden University Medical Centre, Division of Paediatric Endocrinology, Department of Paediatrics, Leiden, The Netherlands

**Keywords:** adrenal insufficiency, adolescents, transition, dutch model

## Abstract

Transition from paediatric to adult healthcare presents unique challenges for adolescents with chronic conditions such as adrenal insufficiency (AI). This process requires careful coordination to ensure continuity of care and support as young patients adapt to managing their condition independently. In the Netherlands, transition care follows a structured, quality-driven approach aimed at meeting the medical, psychological, and social needs of adolescents with chronic conditions. This paper will define key transition-related terms, explain the framework’s five core pillars, explore best practices for transition, and discuss quality indicators and an implementation plan to facilitate effective transition care for AI patients.

## Introduction

The adrenal glands play a crucial role in maintaining homoeostasis by producing essential hormones, including cortisol and aldosterone. Adrenal insufficiency (AI) is a condition characterised by the insufficient production or action of these hormones, leading to significant metabolic, cardiovascular, and immune dysfunctions (1). AI can be classified as primary, secondary, or tertiary, depending on the underlying aetiology, with Addison’s disease being the most common cause of primary AI in adults (2). In children, the most common cause of AI is congenital adrenal hyperplasia (CAH).

In general, adolescence poses several challenges in the management of chronic or complex conditions. Adolescents may show reduced adherence to treatment regimens due to a desire for autonomy, limited insight into long-term health consequences, or discomfort with medical supervision. Hormonal changes and psychosocial development during puberty can further impact motivation, emotional stability, and engagement with care. Issues such as body image, peer pressure, and emerging identity may lead to reluctance in attending follow-up appointments or discussing sensitive topics. These factors underscore the need for developmentally appropriate communication, psychological support, and shared decision-making to optimise care during this critical phase, which has to be embedded during the whole transition process.

Adolescents with AI require lifelong glucocorticoid replacement therapy and stress-dose management to prevent adrenal crises, a life-threatening emergency resulting from acute cortisol deficiency. Transition from paediatric to adult care is a critical period for these patients, as gaps in management and adherence to medical treatment can significantly increase the risk of adverse outcomes, including adrenal crises and hospitalisations. Despite the well-established need for structured transition care, many adolescents experience discontinuity in healthcare services, leading to poor disease control and increased morbidity.

In the Netherlands, a structured approach to transition care for adolescents with AI has been developed to ensure continuity and optimise long-term health outcomes. This well-coordinated, multidisciplinary transition programme, which involves endocrinologists, paediatricians, and specialised nursing teams, can help mitigate risks associated with non-adherence and inadequate stress-dose adjustments during illness, surgery, or psychological stress. Establishing standardised protocols and educating both patients and caregivers about adrenal crisis (AC) prevention are critical components of effective transition care (3).

The Dutch transition care module was developed as part of the national adrenal care pathway by national authorities in collaboration with patient organisations. The transition module was prepared by a healthcare provider specialised in adrenal conditions (HC), together with a process coordinator and patient representative. The concept was sent to the Dutch paediatric and adult endocrinology societies for feedback. The final version was approved by both organisations. For the implementation in healthcare settings, all hospitals treating adolescents and young adults with AI received the transition module, along with a recommendation to organise care accordingly. Hospitals were asked to include the steps towards transition readiness on their own websites or to refer to the adrenalNET website, where the ‘Care Pathway for Adrenal Insufficiency Transition’ was made available. The module was presented at different meetings and sent to the Dutch Society for Endocrinology and the National Working Group of Endocrinology Nurses, with a request for publication on their respective websites.

In this paper, we highlight this structured transition care model in the Netherlands for adolescents with AI, emphasising the need for tailored interventions to prevent care gaps and ensure optimal disease management.

## Defining key transition-related terms and transition programmes

Transition of care is defined as a structured, planned process wherein the medical, psychosocial, educational, and developmental needs of adolescents and young adults with a chronic illness or physical or mental limitations are addressed to facilitate a well-coordinated and continuous care provision adjusted to the phase in development.

The transition phase is a period in which the adolescent receives gradually more responsibilities for his/her own disease and health, and in which independence and self-management of the adolescent increase. Due to the transition process, the adolescent and young adult will be better equipped to move from child-oriented to adult-oriented care (4). The transition process starts early in adolescence and continues after the actual transfer to adult services (5, 6, 7).

Transition readiness is defined as the extent of knowledge and skills of the adolescent and/or young adult to make the transfer to adult healthcare. Transition readiness depends on several factors, including the adolescents’ (pubertal) development, emotional maturity, health status, environment, and school career. The transfer to adult care service should be postponed to a stable situation regarding these factors. In addition, transition readiness is independent of the adolescents’ biological age. According to Dutch law, a young person is an adult from the biological age of 18, but biological age is not a good measure of the adolescents’ or young adults’ mental transition maturity.

Various transition programmes and tools are available to determine transition readiness. In many centres in the Netherlands, the Ready Steady Go (RSG) programme is used for this purpose (https://www.readysteadygo.net/home.html). The RSG programme is a general, non-disease-specific tool developed for adolescents from 12 years onwards and young adults with a chronic illness. It focuses on several domains: knowledge, advocacy, health and lifestyle, daily living, education and the future, work and leisure, and managing emotions. Other available transition tools assessing transition readiness and patient empowerment are the Transition Readiness Assessment Questionnaire (TRAQ), the Gothenburg Youth Patient Empowerment Scale (GYPES), and the Transition-Q questionnaire.

TRAQ (https://www.etsu.edu/com/pediatrics/traq/) was developed for youth 14–21 years of age and focuses on the domains managing medications, appointment keeping, tracking health issues, and talking with providers. The GYPES measures empowerment of adolescents in the transition phase (8).

Transition-Q measures self-management skills for health and disease in chronic conditions (9). For adolescents and young adults with AI, specific criteria for transition readiness are the following: a) substantial knowledge about AI; b) adequate self-management and self-reliance in daily life and in situations when stress management is needed; c) ability to take responsibility for management of medication and outpatient consultations; d) being able to make decisions on their own.

## The five core pillars of transition care framework

**Figure 1:**
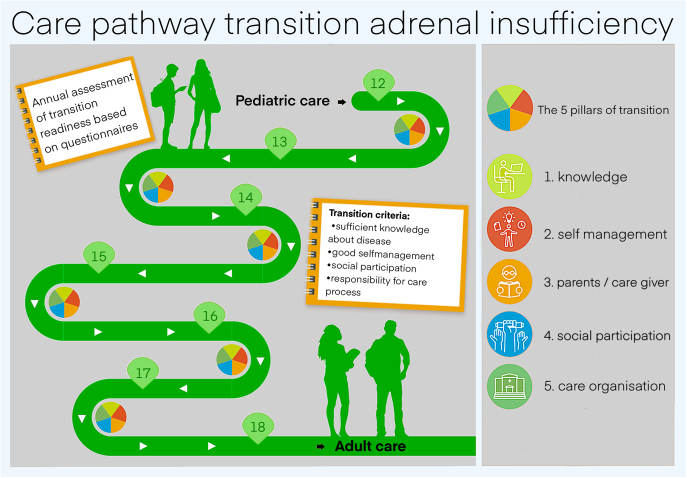
The five core pillars of transition care framework (based on the BijnierNET ‘Zorgpad Transitie bijnierschorsinsuffiëntie’ infographic: https://www.bijniernet.nl/wp-content/uploads/2021/06/Zorgpad-Transitie-bijnierschorsinsuffientie-_met-teller.pdf).

Transition is most successful when not viewed as a one-time event, but as a process that occurs/spans over many years (10). The entire transition process consists of four steps, namely preparation, planning, transfer, and integration in adult care. In the Dutch transition model for adrenal diseases, five pillars for transition care are established (Fig. 1).

### 1. Increasing knowledge in adolescents and parents/caregivers

Possessing adequate knowledge about the condition and its treatment forms the keystone for developing self-management skills in adolescents. In addition, it is essential to understand the disease background and its treatment. Having sufficient knowledge about the treatment of AI and the risk of developing AC can reduce the risk of short- and long-term complications. The gradual introduction of knowledge about the condition and its treatment can commence at an early age (around 12 years or even earlier) during outpatient clinic visits. Research on young people’s understanding of their chronic condition indicates that achieving an adequate level of knowledge contributes to their readiness for transition (11, 12, 13). Involving adolescents in learning about their condition is crucial; however, when parents also possess sufficient knowledge, it positively influences the adolescent’s understanding, strengthens parent-child interactions, and enhances the adolescent’s self-management skills (13).

### 2. Promoting self-management in/by adolescents

Promoting self-management during the transition from paediatric to adult care is essential to ensure adolescents become empowered and capable of managing their own health as they enter adulthood. This transition should be gradual and well-structured, providing adolescents with the tools, resources, and support needed to take increasing responsibility for their care. Introducing self-management support early – typically around ages 12 to 14 – and progressively increasing involvement fosters confidence, autonomy, and readiness for adult care. This includes understanding their diagnosis, treatment options, medication adherence, and making and attending appointments by themselves. Healthcare providers should adopt the role of a coach, supporting self-management by assessing and responding to each adolescent’s motivations. This approach enables the development of a personalised transition plan that aligns with the individual’s knowledge, skills, and readiness for adult care (14). For identifying knowledge and motivations, various tools are available, which were described in the previous section. These tools are designed to assess and support specific aspects of an adolescent’s capabilities or readiness in the transition phase. Overall, the TRAQ and similar questionnaires are seen as valuable tools that can be used for any chronic conditions. It is supposed to lead to improved clinical outcomes, enhanced quality of life (QoL), and better alignment between patient and caregiver perspectives during the transition phase (11, 15).

### 3. Defining appropriate key roles for parent(s)/caregivers(s)

Parents and caregivers play a vital role throughout the various phases of their child’s healthcare transition. For years, they have managed responsibilities such as scheduling appointments, administering medications, and accompanying their child to hospital visits. As the child matures, parents – alongside healthcare providers – should gradually guide them toward greater independence. To support this shift, parents need guidance in transitioning from being the primary managers of their adolescent’s health to becoming facilitators who encourage and support their adolescent’s growing autonomy (16). Guiding adolescents toward independence can create tension for parents, who must balance being vigilant about their child’s progress with learning to trust and let go. A qualitative study involving adolescents with chronic conditions highlights differing perceptions between adolescents and their parents on key themes such as independent living, intimate relationships, leisure activities, and healthcare. Professional support in these areas may help adolescents strengthen their autonomy while also reducing parent-child conflict and mitigating potential negative outcomes (17). Parents can support their child in the transition process by actively discussing what it means to have a chronic condition and teaching them how to manage their own medical condition. Parental involvement during transition and after transfer to adult care was found to have a positive impact on patients’ transition experiences overall (18). Specific questionnaires for parent(s) and caregiver(s) from the RSG Programme can help the multidisciplinary team to initiate conversations with parents about their role in the transition period and identify gaps in knowledge and skills.

### 4. Social participation

Participation is defined by the WHO as ‘involvement in a life situation’, such as engaging in social interactions or taking on a role in sports or academia (WHO, 2002). From the onset of transition care in the paediatric setting, adolescents and young adults often experience significant social changes, such as completing secondary school, moving out of the family home, starting university, or entering the workforce. Active participation in daily life is crucial for their development and plays a key role in supporting a healthy and successful transition into adulthood (19). A Dutch study among adolescents with a chronic condition reveals these children have lower social participation compared to healthy peers. Adolescents with a chronic condition rate their QoL lower, are less frequently members of sports clubs, and indicate a preference for working fewer hours per week. Social participation depends on child factors but also on the disease and its impact on daily life (20). Children with CAH experience several daily health-related problems, but these do not hamper them in their daily activities and participation in society (21). Nevertheless, it remains important to address different aspects of social participation during transition. According to the WHO definition of participation, social participation is not only about participating in social activities, but the sense of belonging is as important. Focusing on full participation from the child’s perspective will facilitate patient-centred care by helping the child self-manage their participation (22).

#### Risk behaviour

A specific aspect of social participation in adolescents with chronic conditions is engagement in risk behaviours, such as the use of tobacco, alcohol, and drugs. Like their peers, adolescents with chronic illnesses – such as AI – may encounter these substances. However, it is especially important for those with AI to discuss such behaviours with their healthcare providers. These conversations can raise awareness about the potential health consequences and help adolescents understand the precautions necessary to prevent medical emergencies. For adolescents with AI, it is important that they inform friends about their condition and enable them to explain to emergency healthcare providers what condition the adolescent or young adult has in case of emergencies. In addition, it is advisable that at least one of the friends remains capable of acting appropriately during emergencies. In such situations, it is important to wear an SOS card or to use an app that provides instructions on what to do in medical emergencies.

### 5. Organising care to align with the adolescent’s needs

A purposeful and planned movement from paediatric to adult care is essential for ensuring a smooth and effective transition. This process requires structuring healthcare services that not only accommodate the medical complexity of AI but also cater to the developmental stage of the adolescent. The transition must be patient-centred and responsive to the needs of the adolescent, and as such, healthcare providers must work collaboratively with families and patients.

A cornerstone of this approach is the coordination between paediatric and adult endocrinology teams to ensure continuity of care. This collaboration should involve regular communication and joint consultations to facilitate a seamless transition. Studies have demonstrated that multidisciplinary transition clinics, where paediatric and adult specialists collaborate, can significantly improve outcomes by providing consistent, coordinated care (23).

The paediatric endocrinologist usually coordinates early medical management and initiates the transition process. The case manager, often a specialised nurse, supports care coordination and patient education throughout the transition. A psychologist is involved early to address emotional and developmental needs, while a sexologist provides guidance on sexuality, body image, and relationships, particularly during and after puberty. The adult endocrinologist is introduced before transfer to ensure continuity of care and to establish trust, ideally through joint consultations during the transition phase.

Another well-established intervention in organising care for adolescents transitioning from paediatric to adult care is a transition coordinator. Transition coordinators help bridge the gap between paediatric and adult care. The role of the transition coordinator is ensuring that adolescents receive appropriate guidance, managing appointments and tracking medical records, and offering support and resources throughout the transition process. This role has been shown to improve the overall quality of care and support for both the adolescent and their family (24).

Transition of adolescents with chronic conditions is similar across various diseases; however, there are disease-specific considerations for adolescents with different forms of AI. For example, as adolescents with CAH progress through puberty, issues related to fertility and sexual function become more prominent and necessitate focused attention (25). Regular monitoring for gonadal dysfunction and menstrual irregularities is becoming an essential component of routine clinical care. Besides the paediatric and adult endocrinologist, it is recommended to have a coordinated approach involving other specialists, including urologists or gynaecologists, and psychologists or psychiatrists.

## Dutch best practices in transition care of patients with AI

Adherence to glucocorticoid replacement therapy is a fundamental aspect in the effective management of AI. However, during the transition phase, challenges arise that may impact adherence. Adolescents often face difficulties in independently managing their treatment regimen due to both developmental factors and the increasing responsibility for their own healthcare. At the same time, parents/caregivers must adapt by stepping back, allowing the adolescent to take on this responsibility themselves. This requires a delicate balance of support and independence.

### Case report

A 20-year-old university student with CAH was diagnosed with salt-wasting CAH during the neonatal period and treated with hydrocortisone and fludrocortisone. He was followed by the paediatric team, and from 12 years onwards, he had regular separate appointments with the specialised nurse following the transition plan. He was referred to the adult endocrinologist at the age of 19 years after he started his study and moved to his study place. He attends a house party with several fellow students. A week before the party, he returned from a holiday with his parents. Shortly before arriving home, he fell ill and was briefly hospitalised due to an AC, the cause of which was unknown. After his hospital stay, he recovered, but at the party, he again felt unwell and was on the verge of losing consciousness. He tried to alert his friends that he was sick, but they were unable to respond adequately due to excessive alcohol consumption. The young adult decided to call the emergency number himself. The ambulance arrived, and he received an emergency hydrocortisone injection. He was able to recognise the symptoms of an AC and, despite his friends being unable to help, he managed to call the ambulance himself. A week later, he was hospitalised again and was diagnosed with appendicitis.

Patients with AI usually take medication multiple times per day. During infections with fever, times of severe stress, and during surgical interventions, all individuals with AI are at risk of developing AC if not treated adequately (26). Patient education is regarded as a key factor contributing to preventing adrenal crises (26). Regularly repeated educational sessions on AI, with an emphasis on its symptoms, stress management strategies, and the critical importance of medication adherence should be part of routine clinical visits. To connect with the target group, age-appropriate educational materials that resonate with adolescents could make the information more relatable and therefore easier to understand (https://rise.articulate.com/share/hIhomowQzuTVlDcTLEmMdSroQBn4t43p#/). Practical education on the emergency injection technique is also an important part of the educational sessions.

Today, a modified-release hydrocortisone preparation is available for patients with CAH, which may improve therapy adherence by allowing twice-daily dosing. However, the current literature does not yet provide clear evidence to confirm this potential benefit.

We stimulate the use of mobile applications or digital information to empower adolescents to manage their condition independently. There are indications that internet- and mobile-based interventions might improve self-efficacy and disease-related somatic outcomes in paediatric patients (27). Domhardt *et al.* point out a rather small benefit and limited efficacy. The use of digital tools (e.g. smartphone apps, patient portals) helping adolescents track their health status and communicate with providers is not proven. There are indications that internet- and mobile-based interventions improve self-efficacy and disease-related somatic outcomes in paediatric patients. However, Domhardt *et al.* point out a rather small benefit and limited efficacy. Nevertheless, it is confirmed that AYAs were receptive to receiving medical information electronically (28).

## Parameters/outcome measures for effective transition

AC most commonly occurs during episodes of gastroenteritis or other infections, often leading to emergency room visits that require intravenous glucocorticoid administration. While comprehensive studies are lacking for many types of AI, AC is recognised as a life-threatening complication. In patients with CAH, AC has been associated with increased mortality (29). In retrospective observational studies, AC occurs 3.8–6.6 times per 100 patient years in patients with primary AI (30). A recent prospective German study found that AC was more common in adults (8.4 episodes per 100 PY) than in children (5.1) with CAH. The same held true for the need for stress dosing (190 episodes per 100 PY vs 175) (31). In Australia, the incidence of AC has increased over the first two decades of this century (32). There was a disproportionate increase in hospital admissions in women aged 20–29 years, likely related to psychosocial factors (33). In a German cohort, Reisch and colleagues noticed a peak in AC episodes among patients aged 18–25 years, followed by a decline at older ages, suggesting that the post-transition age is a particularly vulnerable life stage regarding the occurrence of AC (34).

## Patient-reported outcomes including self-efficacy and treatment satisfaction

Knowledge of and adherence to the treatment recommendations have been reported to be lower in the adult compared with the paediatric AI population, emphasising the need for structured and repeated education (31). However, so far interventions for the prevention of AC have not resulted in dramatic reductions of AC events (35). Two Dutch studies showed conflicting results: one or two educational sessions were not effective to achieve adequate self-management skills in a group of AI patients (36). In contrast, Repping *et al.* found that glucocorticoid education groups improved self-management aimed at preventing adrenal crises (37).

QoL is affected in AI patients with CAH (38). Good medication adherence was found to be associated with better transition readiness, and good transition readiness was associated with increased QoL scores (39). Bachelot *et al.* found that compliance with regular medical follow-up in adulthood was associated with the transition phase and with better QoL in adults with CAH, but not specified for AI aspects (40). There is no published literature on patient-reported outcome measures (PROMS) in patients with AI.

## Assessment of healthcare provider communication and coordination

Loss to follow-up is common during and after the transition period (5). This may not be prevented by a successful transition. In a period of 20 years in a tertiary referral centre for AI, Kiewert *et al.*, reported that 40.7% of patients were lost to follow-up despite a successful transition for the majority of the patients in the clinic (41). A recent Dutch study found a lower percentage of loss to follow-up, possibly due to the presence of a case manager/nurse specialist who guides patients with CAH through the transition process (42). The authors suggested that a structured handover protocol may prevent dropouts. Structured communication with caregivers could lead to quality improvement of AI patient care (43). As such, a strategy could be readily adopted by caregivers; structured communication should be a feasible outcome measure. Future indicator sets for AI could be adapted from more generic indicator sets for transition in young adults with chronic conditions (44).

## Quality criteria and quality indicators

Quality criteria should be guaranteed for adequate transition care for all chronic conditions. Quality indicators provide valuable quality information for both healthcare providers and patients and their families. Documentation based on these indicators ensures transparency. Both quality criteria and indicators for AI are summarised in Table 1.

**Table 1:** Quality criteria and quality indicators for adequate transition care for individuals with adrenal insufficiency.

Category	Description
Quality criteria	Transition care must be provided in a centre with a multidisciplinary team experienced in adrenal disorders
	Treating paediatric and adult endocrinologists must adhere to current clinical guidelines for diagnosis and treatment
	Healthcare providers must remain up to date with emerging scientific evidence and best practices
	The transition team should include at least a paediatric endocrinologist, an adult endocrinologist, a nurse specialist, and, when appropriate, a psychologist
	The transition process must be transparent, with active involvement of the patient and their parents or caregivers
	A personalised transition plan should be developed, considering individual readiness, personal circumstances, and preferences
	Coordination must be maintained throughout the entire transition period
	Paediatric and adult endocrinologists should provide regular written updates to the general practitioner
	Patient education should be tailored to the patient’s age, maturity, and preferences
	The adrenal care team, including endocrinologists and nurse specialists, must be accessible 24/7
Quality indicators	Annual consultation with a nurse specialist
	Annual written communication to the general practitioner, including stress dosing instructions and medical updates
	Dropout rate at transition to adult care should be <1%
	Emergency department visits related to adrenal insufficiency should be <1% annually

## Implementation from the patients’ perspective

To ensure a successful and structured implementation of the transition care quality module for adolescents and young adults with AI, a comprehensive approach has been designed. This plan focuses on healthcare providers, young patients, their families, and the wider community.

The first area of implementation targets healthcare providers. All hospitals involved in the treatment of young individuals with AI will receive the quality module, along with a recommendation to organise their services according to its guidelines. To enhance professional preparedness, specialised training modules should be developed and offered to healthcare providers. These modules will focus on managing adrenal crises during the transition period, a critical skillset for ensuring continuity and safety in care. Furthermore, a stepwise approach tailored specifically to AI will be implemented. This includes clearly defined milestones for developing self-management skills among adolescents, such as understanding their condition, recognising early warning signs, and managing medication independently. These milestones will guide healthcare professionals and families through each phase of the transition (Fig. 1).

The second component of the plan focuses on adolescents, young adults, and their caregivers. Recognising the essential role of family, the plan includes engagement strategies aimed at helping parents support their children’s growing independence. Parents will be guided on how to gradually transfer responsibility while maintaining safety and emotional support.

Patient support groups will also play an important role in the transition process. These groups can provide peer support, education, and a sense of community for adolescents and their families. They can help normalise the challenges of living with AI, offer practical advice, and reduce the emotional burden often associated with chronic illness. Involvement in support groups may enhance motivation for self-management and empower young people by connecting them with others facing similar experiences.

Beyond the healthcare setting, coordination with schools and workplaces will be encouraged. This is to ensure that adolescents and young adults with AI receive appropriate accommodations and support, especially in managing their medical needs in educational and professional environments.

Supporting self-management is a cornerstone of this plan. Adolescents should be encouraged to take shared responsibility for their care, participate actively in regular consultations, and prepare for medication adjustments. Parents will be supported in developing trust in their child’s ability to manage their condition. The use of eHealth technologies will be promoted as tools to empower young people, and healthcare providers are advised to consistently reinforce stress management instructions.

This comprehensive and inclusive implementation plan is designed to guide young individuals with AI – and everyone involved in their care – through a safe, supported, and confident transition from paediatric to adult healthcare services.

## Discussion

Transition from child-centred care to adult-centred care is a critical period for the adolescent’s health and well-being. While the actual transfer is only a brief phase, the phase ahead of the transfer and the aftercare is of major importance to prevent loss to follow-up, medication mistakes, and insufficient advocacy of their own health and well-being.

In AI, the risk for AC is a burden, making the whole process of transition and awareness even more important. Many adolescents are reluctant to be open about their medical condition to friends and peers because of fear of being different or not being accepted. This illustrates the challenges specific to AI: in case of an emergency, help from a friend can be crucial for adequate medical treatment/emergency medication. During transition, situations such as going to a party, the pitfalls of using alcohol or drugs, or going on holiday with friends without the parents being present are discussed. During these conversations, situations are practised, and adolescents are supported in exposing their needs to friends and peers.

Potential barriers during the transition phase include the lack of a dedicated transition coordinator to track records and ensure appointments are scheduled after transfer to adult care. Ideally, transition to an adult endocrinologist in the same hospital starts with combined consultations. During these combined consultations, the adolescent and their parents can get acquainted with their new physician. Differences in child- and adolescent- adrenal care can be discussed in the early phase and again during these combined consultations. For example, differences in available glucocorticoids before and after 18 years of age will be provided, and updates on age-adjusted sick-day rules will be explained. In this way, the adolescent will be better prepared to take care of his/her own health and disease throughout the whole transition phase until early adulthood.

The prevalence and presentation of adrenal conditions differ significantly between children, adolescents, and adults. While, for example, adrenocortical carcinoma is an extremely rare condition in childhood, it should always be considered in the differential diagnosis of children presenting with signs of adrenal disease. Conversely, congenital adrenal conditions are more common during childhood. It is essential that both paediatric and adult endocrinologists are aware of these differing spectrums and continue to learn from one another, particularly with regard to diagnostic evaluation. The transition from paediatric to adult care provides an excellent opportunity to exchange perspectives and ensure continuity of expertise across age groups.

This smooth transition maintains confidence in the medical team, while an abrupt change in medication or treatment plan will undermine the trustworthiness of the medical team.

Another potential barrier in the transition to adult services might be a lack of resources. For example, in the Netherlands, from 18 years onwards, each individual needs to pay for their health insurance. Medical visits are covered by insurance; however, part of the costs for medication/medical visits must be paid by the patient (maximised at ∼360 euro each year). For young adults, this amount might be a potential barrier to scheduling appointments.

Another potential barrier is the different focus during medical consultations in adult medicine, i.e. there might be more focus on the medical condition and less on the person themselves. Most studies have investigated transition in patients with CAH. Information about transition in other adrenal conditions is scarce. Future research should focus on the broad spectrum of adrenal conditions and the special needs of patients with these conditions in daily life. For example, participation in sports, education, work, and interaction with peers. What do patients need from the medical team, what should they be prepared for, and what do they need to learn during different phases?

## Conclusion

In conclusion, the transition from child- to adult-centred care in adolescents with AI is a complex and vulnerable period that requires careful planning and support. The risk of AC, social stigma, and system barriers such as insurance costs and access to different types of medication all contribute to the challenges faced. A well-structured, tailored transition is essential to ensure continuity of care, patient safety, and empowerment.

This underscores the importance of transition care that is specifically designed for the needs of adolescents with AI. A multidisciplinary, patient-centred approach – bringing together endocrinologists, nurses, psychologists, and social workers – can help address both medical and psychosocial challenges.

To improve outcomes, it is crucial for healthcare providers, institutions, and policymakers to invest in structured transition programmes that offer continuity, education, and emotional support. These efforts are key to helping adolescents confidently manage their condition and thrive into adulthood.

## Declaration of interest

The authors declare that there is no conflict of interest that could be perceived as prejudicing the impartiality of the work reported.

## Funding

This work did not receive any specific grant from any funding agency in the public, commercial, or not-for-profit sector.

## References

[1] Bornstein SR, Allolio B, Arlt W, et al. Diagnosis and treatment of primary adrenal insufficiency: an endocrine society clinical practice guideline. J Clin Endocrinol Metab 2016 101 364–389. (10.1210/jc.2015-1710) 26760044 PMC4880116

[2] Husebye ES, Allolio B, Arlt W, et al. Consensus statement on the diagnosis, treatment and follow-up of patients with primary adrenal insufficiency. J Intern Med 2014 275 104–115. (10.1111/joim.12162) 24330030

[3] Speiser PW, Arlt W, Auchus RJ, et al. Congenital adrenal hyperplasia due to steroid 21-hydroxylase deficiency: an endocrine society clinical practice guideline. J Clin Endocrinol Metab 2018 103 4043–4088. (10.1210/jc.2018-01865) 30272171 PMC6456929

[4] Blum RW, Garell D, Hodgman CH, et al. Transition from child-centered to adult health-care systems for adolescents with chronic conditions. A position paper of the Society for Adolescent Medicine. J Adolesc Health 1993 14 570–576. (10.1016/1054-139x(93)90143-d) 8312295

[5] Gleeson H, Davis J, Jones J, et al. The challenge of delivering endocrine care and successful transition to adult services in adolescents with congenital adrenal hyperplasia: experience in a single centre over 18 years. Clin Endocrinol 2013 78 23–28. (10.1111/cen.12053) 23009615

[6] Gleeson H & Turner G. Transition to adult services. Arch Dis Child Educ Pract Ed 2012 97 86–92. (10.1136/archdischild-2011-300261) 21979963

[7] Gleeson H, McCartney S & Lidstone V. Everybody’s business’: transition and the role of adult physicians. Clin Med 2012 12 561–566. (10.7861/clinmedicine.12-6-561) PMC5922597 23342411

[8] Acuna Mora M, Luyckx K, Sparud-Lundin C, et al. Patient empowerment in young persons with chronic conditions: psychometric properties of the Gothenburg young persons empowerment scale (GYPES). PLoS One 2018 13 e0201007. (10.1371/journal.pone.0201007) 30028863 PMC6054395

[9] Klassen AF, Grant C, Barr R, et al. Development and validation of a generic scale for use in transition programmes to measure self-management skills in adolescents with chronic health conditions: the TRANSITION-Q. Child Care Health Dev 2015 41 547–558. (10.1111/cch.12207) 25351414

[10] Merke DP & Poppas DP. Management of adolescents with congenital adrenal hyperplasia. Lancet Diabetes Endocrinol 2013 1 341–352. (10.1016/s2213-8587(13)70138-4) 24622419 PMC4163910

[11] van Gaalen MAC, van Gijn E, van Pieterson M, et al. Validation and reference scores of the transition readiness assessment questionnaire in adolescent and young adult IBD patients. J Pediatr Gastroenterol Nutr 2023 77 381–388. (10.1097/mpg.0000000000003868) 37347146

[12] van Gaalen MAC, van Pieterson M, van den Brink G, et al. Rotterdam transition test: a valid tool for monitoring disease knowledge in adolescents with inflammatory bowel disease. J Pediatr Gastroenterol Nutr 2022 74 60–67. (10.1097/mpg.0000000000003278) 34371508

[13] Stewart KT, Chahal N, Kovacs AH, et al. Readiness for transition to adult health care for young adolescents with congenital heart disease. Pediatr Cardiol 2017 38 778–786. (10.1007/s00246-017-1580-2) 28184978

[14] Williams ES, Enzler CJ, Bretz L, et al. Development of self-management skills in 14- to 16-year-old adolescents with chronic health conditions: a qualitative study. Child Care Health Dev 2024 50 e70012. (10.1111/cch.70012) 39569805

[15] Takeuchi J, Yanagimoto Y, Sato Y, et al. Efficacious interventions for improving the transition readiness of adolescents and young adult patients with chronic illness: a narrative review of randomized control trials assessed with the transition readiness assessment questionnaire. Front Pediatr 2022 10 983367. (10.3389/fped.2022.983367) 36245732 PMC9554476

[16] Bratt EL, Burstrom A, Hanseus K, et al. Do not forget the parents-parents’ concerns during transition to adult care for adolescents with congenital heart disease. Child Care Health Dev 2018 44 278–284. (10.1111/cch.12529) 28980341

[17] Peeters MA, Hilberink SR & van Staa A. The road to independence: lived experiences of youth with chronic conditions and their parents compared. J Pediatr Rehabil Med 2014 7 33–42. (10.3233/prm-140272) 24919936

[18] Badour B, Bull A, Gupta AA, et al. Parental involvement in the transition from paediatric to adult care for youth with chronic illness: a scoping review of the North American literature. Int J Pediatr 2023 2023 9392040. (10.1155/2023/9392040) 38045800 PMC10691897

[19] King G, McDougall J, Dewit D, et al. Predictors of change over time in the activity participation of children and youth with physical disabilities. Child Health Care 2009 38 321–351. (10.1080/02739610903237352) 19907673 PMC2774928

[20] Martinez W, Carter JS & Legato LJ. Social competence in children with chronic illness: a meta-analytic review. J Pediatr Psychol 2011 36 878–890. (10.1093/jpepsy/jsr035) 21745809

[21] Sanches SA, Wiegers TA, Otten BJ, et al. Physical, social and societal functioning of children with congenital adrenal hyperplasia (CAH) and their parents, in a Dutch population. Int J Pediatr Endocrinol 2012 2012 2. (10.1186/1687-9856-2012-2) 22300447 PMC3292980

[22] Nap-van der Vlist MM, Kars MC, Berkelbach van der Sprenkel EE, et al. Daily life participation in childhood chronic disease: a qualitative study. Arch Dis Child 2020 105 463–469. (10.1136/archdischild-2019-318062) 31748222

[23] Crowley R, Wolfe I, Lock K, et al. Improving the transition between paediatric and adult healthcare: a systematic review. Arch Dis Child 2011 96 548–553. (10.1136/adc.2010.202473) 21388969

[24] Kelly D. Theory to reality: the role of the transition nurse coordinator. Br J Nurs 2014 23 888, 890, 892-884. (10.12968/bjon.2014.23.16.888) 25203759

[25] Balagamage C, Arshad A, Elhassan YS, et al. Management aspects of congenital adrenal hyperplasia during adolescence and transition to adult care. Clin Endocrinol 2024 101 332–345. (10.1111/cen.14992) 37964596

[26] Rushworth RL, Chrisp GL, Bownes S, et al. Adrenal crises in adolescents and young adults. Endocrine 2022 77 1–10. (10.1007/s12020-022-03070-3) 35583847 PMC9242908

[27] Domhardt M, Schröder A, Geirhos A, et al. Efficacy of digital health interventions in youth with chronic medical conditions: a meta-analysis. Internet Interv 2021 24 100373. (10.1016/j.invent.2021.100373) 33732626 PMC7941178

[28] Low JK & Manias E. Use of technology-based tools to support adolescents and young adults with chronic disease: systematic review and meta-analysis. JMIR Mhealth Uhealth 2019 7 e12042. (10.2196/12042) 31322129 PMC6670279

[29] Falhammar H, Frisen L, Norrby C, et al. Increased mortality in patients with congenital adrenal hyperplasia due to 21-hydroxylase deficiency. J Clin Endocrinol Metab 2014 99 E2715–E2721. (10.1210/jc.2014-2957) 25279502

[30] Smans LC, Van der Valk ES, Hermus AR, et al. Incidence of adrenal crisis in patients with adrenal insufficiency. Clin Endocrinol 2016 84 17–22. (10.1111/cen.12865) 26208266

[31] Tschaidse L, Wimmer S, Nowotny HF, et al. Frequency of stress dosing and adrenal crisis in paediatric and adult patients with congenital adrenal hyperplasia: a prospective study. Eur J Endocrinol 2024 190 275–283. (10.1093/ejendo/lvae023) 38584334

[32] Chrisp GL, Quartararo M, Torpy DJ, et al. Trends in hospital admissions for adrenal insufficiency in adolescents and young adults in the 21(st) century. Front Endocrinol 2022 13 986342. (10.3389/fendo.2022.986342) PMC9530131 36204108

[33] Rushworth RL, Falhammar H & Torpy DJ. Factors underlying a disproportionate increase in hospital admissions for adrenal insufficiency in women aged 20–29 years. Front Endocrinol 2023 14 1252577. (10.3389/fendo.2023.1252577) PMC10656680 38027206

[34] Reisch N, Willige M, Kohn D, et al. Frequency and causes of adrenal crises over lifetime in patients with 21-hydroxylase deficiency. Eur J Endocrinol 2012 167 35–42. (10.1530/eje-12-0161) 22513882

[35] Shepherd LM, Schmidtke KA, Hazlehurst JM, et al. Interventions for the prevention of adrenal crisis in adults with primary adrenal insufficiency: a systematic review. Eur J Endocrinol 2022 187 S1–S20. (10.1530/eje-21-1248) 35536876 PMC9175553

[36] van der Meij NT, van Leeuwaarde RS, Vervoort SC, et al. Self-management support in patients with adrenal insufficiency. Clin Endocrinol 2016 85 652–659. (10.1111/cen.13083) 27063934

[37] Repping-Wuts HJ, Stikkelbroeck NM, Noordzij A, et al. A glucocorticoid education group meeting: an effective strategy for improving self-management to prevent adrenal crisis. Eur J Endocrinol 2013 169 17–22. (10.1530/eje-12-1094) 23636446

[38] Arlt W, Willis DS, Wild SH, et al. Health status of adults with congenital adrenal hyperplasia: a cohort study of 203 patients. J Clin Endocrinol Metab 2010 95 5110–5121. (10.1210/jc.2010-0917) 20719839 PMC3066446

[39] Ekbom K, Lajic S, Falhammar H, et al. Transition readiness in adolescents and young adults living with congenital adrenal hyperplasia. Endocr Pract 2023 29 266–271. (10.1016/j.eprac.2023.01.010) 36693541

[40] Bachelot A. Transition of care from childhood to adulthood: congenital adrenal hyperplasia. Endocr Dev 2018 33 17–33. (10.1159/000487523) 29886487

[41] Kiewert C, Jedanowski J, Hauffa BP, et al. Transition from paediatric to adult care in CAH: 20 Years of experience at a tertiary referral center. Horm Metab Res 2024 56 45–50. (10.1055/a-2201-6548) 38171370

[42] Davidse K, van Staa A, Geilvoet W, et al. We mind your step: understanding and preventing drop-out in the transfer from paediatric to adult tertiary endocrine healthcare. Endocr Connect 2022 11 e220025. (10.1530/ec-22-0025) 35521816 PMC9175586

[43] Dennis J, Pitts L, Matalka L, et al. Comprehensive adolescent healthcare transition program for congenital adrenal hyperplasia: a quality improvement initiative. Health Care Transit 2024 2 100057. (10.1016/j.hctj.2024.100057) 39712594 PMC11658564

[44] Sattoe JNT, Hilberink SR & van Staa A. How to define successful transition? An exploration of consensus indicators and outcomes in young adults with chronic conditions. Child Care Health Dev 2017 43 768–773. (10.1111/cch.12436) 28074484

